# Effectiveness of Ozone Treatment and Packaging Techniques in Preserving Taiwanese Domestic Beef During Refrigerated Storage

**DOI:** 10.3390/foods13213471

**Published:** 2024-10-30

**Authors:** Chao-Wei Huang, Shiro Takeda, Yen-Po Chen, Fu-Yuan Cheng, Pei-Jung Wu, Liang-Chuan Lin, Yu-Tse Liu

**Affiliations:** 1 Department of Tropical Agriculture and International Cooperation, National Pingtung University of Science and Technology, Pingtung 912, Taiwan; cwhuang@mail.npust.edu.tw; 2 School of Veterinary Medicine, Azabu University, Sagamihara 252-5201, Japan; s-takeda@azabu-u.ac.jp; 3 Department of Animal Science, National Chung Hsing University, Taichung City 402, Taiwan; chenyp@dragon.nchu.edu.tw (Y.-P.C.); peigooo@hotmail.com (P.-J.W.); chuan4354@hotmail.com (L.-C.L.); 4 Department of Animal Science, National Pingtung University of Science and Technology, Pingtung 912, Taiwan; fycheng@mail.npust.edu.tw

**Keywords:** ozone, PVDC packaging, vacuum packaging, domestically produced beef, antibacterial

## Abstract

This study investigates the efficacy of ozone treatment combined with different packaging methods on the preservation of Taiwanese domestically produced beef during refrigerated storage. The preservation of fresh beef is crucial for ensuring food safety and quality; we do not know whether changing the packaging method can mitigate the negative effects of ozone on meat and even enhance its positive impact. Beef samples were treated with ozone and packaged using the vacuum or PVDC-tray methods, then stored at 4 °C for 7 days. The results show that ozone treatment effectively inhibited microbial (total plate count, *Salmonella*, and *Escherichia coli*) growth (*p* < 0.05). Vacuum packaging maintained lower TBARS values (*p* < 0.05) and metmyoglobin percentages compared to PVDC-tray packaging (*p* < 0.05). The L* values of all treatments increased over storage time, with significant differences observed between days 0 and 7. Ozone treatment combined with vacuum packaging demonstrated promising results in inhibiting microbial growth and preserving beef quality during refrigerated storage. These findings contribute to enhancing the safety and shelf life of Taiwanese domestically produced beef, potentially benefiting both producers and consumers.

## 1. Introduction

Beef, one of the world’s most popular meats, serves as an important source of both macronutrients and micronutrients. The macronutrients consist of water, high-quality protein, and fats that include both saturated and unsaturated fatty acids. In terms of micronutrients, beef provides heme iron, zinc, selenium, and vitamins D, B1, B2, B3, B5, B6, and B12 [1]. However, beef is easy to spoil and loses quality during postharvest handling and storage, which is mainly due to enzymatic and microbial activities that impact beef’s freshness and quality [2]. Although Taiwan’s self-sufficiency rate for beef is less than 5%fresh domestically produced Taiwanese beef still has its consumer market; therefore, we would also like to understand the microbial situation of domestically produced beef in Taiwan and potential techniques for improving its preservation.

Various preservation techniques have been employed to prolong the shelf life and maintain the quality of beef. These methods include freezing, refrigeration, vacuum packaging (VP), modified atmosphere packaging (MAP), and irradiation. However, it is important to acknowledge that each of these methods has specific limitations. These constraints may have an impact on the nutritional and sensory qualities of beef. Additionally, these techniques can be used alone or in combination with one another [2]. In 1997, the Food and Drug Administration (FDA) considered ozone a Generally Recognized as Safe (GRAS) substance in different food applications, which has increased its use worldwide [3,4]. In August 2000, the Food and Drug Administration (FDA) in the U.S.A. approved ozone as a direct additive for treating, storing, and processing foods, in its gas or aqueous phase [5]. Ozone is a powerful disinfectant and oxidant, offering several advantages in its application. Notably, excess ozone rapidly decomposes into oxygen, leaving no harmful residues in food from its breakdown [6,7]. It has a stronger antimicrobial ability than chlorine and is considered a broad-spectrum antimicrobial agent that acts against a variety of foodborne pathogens [8]. The scientific literature reports various results depending on ozone concentrations and application methods. In a study carried out by [9], beef and poultry inoculated with Salmonella were treated with ozone concentrations higher than 10,000 ppm; while this level achieved up to a 99% reduction in microbial populations, it also led to undesirable off-flavors and color changes in the meat. These negative effects restrict the maximum amount of ozone that can be safely applied to food.

Packaging beef helps prevent deterioration. Vacuum-sealed packages, which eliminate oxygen, can reduce oxidative degradation, and extend the meat’s shelf life [10]. Taiwanese domestically produced beef primarily comes from culled dairy cows, which are slaughtered after being fattened. Due to their longer rearing period, the meat tends to have a darker coloration. Vacuum packaging is commonly used during retail, but the absence of oxygen (O_2_) within the packaging prevents the formation of oxymyoglobin (OMb) by oxygenating myoglobin (Mb) in the meat, resulting in a bright red color. This discrepancy in color may lead consumers to mistakenly perceive the meat as lower quality due to its darker appearance, thereby reducing their willingness to purchase it. Polyvinyl chloride (PVC) and Polyvinylidene chloride (PVDC) film, which is used for the retail storage of meat and is extremely permeable to oxygen can, therefore, enhance the appearance of the meat with better coloration, increasing purchasing intention, and it is also the most cost-effective packaging [11].

There is currently no research on the impact of ozone sterilization technology and different packaging methods on the preservation of Taiwanese domestically produced beef. While ozone is a powerful oxidizing agent that can significantly affect the sensory, physical, and chemical properties of meat, its high oxidation potential may primarily contribute to lipid and protein oxidation in the meat [12], but we do not know whether changing the packaging method can mitigate the negative effects of ozone on meat and even enhance its positive impact. Therefore, the goal of this research is to evaluate the effects of ozone water combined with commonly used PVDC-tray packaging or vacuum packaging on the storage characteristics, common pathogenic microorganisms, color appearance, and metmyoglobin content of Taiwanese domestically produced beef during refrigerated storage. The goal is to help improve the preservation and food safety of fresh domestically produced Taiwanese beef during storage and sale.

## 2. Materials and Methods

### 2.1. Raw Beef Meat

Ten culled dairy cattle carcasses were randomly selected from two local slaughterhouses in Taiwan (Taichung and Chia-Yi). The commercial cut conditions applied in this study focused on the top inside round, which includes the adductor femoris and semimembranosus muscles. Each cut weighed approximately 4.0 kg, resulting in a total of 20 beef samples. The muscles were separated 48 h post mortem, with all visible fat trimmed away. The raw materials exhibited a pH value ranging from 5.5 to 5.7. The beef samples were then transported to the laboratory on ice, where they were sectioned into disks measuring 0.5 cm thick and 6 cm in diameter. The beef samples were randomly grouped for experiment treatments, the meat samples analyzed at 5 different times (day 0–day 7) were separated at time zero. All samples underwent experiments with a minimum of three batches and three replicates each.

### 2.2. Ozone Generation and Detector

The corona discharge technique was employed in an ozone generator (Ozonizer, model OZ-8000S, Fuhan Technology Co., Ltd., Taipei, Taiwan). The ozone generator has a production capacity of 2000 mg per hour, and the ozone concentration in water can be maintained between 1 ppm and 2 ppm. Also, the temperature is 23.5 °C with a relative humidity of 75%. It involves applying a high voltage (in the kilovolt range) between two electrodes while passing oxygen or air through them. This process generates an electric shock that breaks oxygen molecules into monoatomic oxygen, recombining with diatomic oxygen to form ozone molecules. Utilizing an ozone detector (AOM-05, Aidenshi Co., Ltd., Nasushiobara, Tochigi-Ken, Japan) to verify that the concentration of ozone water meets the requirements of the experimental design.

### 2.3. PVDC-Trap and Vacuum Packaging

After subjecting beef to either 30 s of ozone treatment (Ozone) or no treatment (Control), two common packaging methods were used. For the PVDC-trap-treated group, Styrofoam trays (135 × 80 × 20, KOUSHO ENTERPRISE Co., Ltd., Taipei City, Taiwan) and PVDC film (permeability: 3 mL/m^2^ × h × atm(STP)@20 °C, 80%RH, KUREHA, Tokyo, Japan) were utilized for the packaging. For the vacuum-treated group, vacuum packaging bags (150 mm × 225 mm × 0.08 mm, NY, Dah Yeou Industrial Co., Ltd., Taiching City, Taiwan) were used and vacuumed to a vacuum level of −95,000 Pa (−0.95 bar) using a vacuum packaging machine (YU-601A, Tabletop Vacuum Packing Machine, UMPACKT, Taichung, Taiwan) before sealing.

### 2.4. Experimental Grouping and Refrigerated Storage Test

C-PVDC = Control + PVDC with tray packaging, C-Vacuum = Control + vacuum packaging, O-PVDC = Ozone water 30 s + PVDC with tray packaging, O-Vacuum = Ozone water 30 s + vacuum packaging. Following packaging, all treatment groups were placed in cold refrigeration at 4 °C (NR-B371TV, Panasonic, Taipei City, Taiwan) for storage testing. Samples were retrieved for various analyses on days 0, 1, 3, 5, and 7.

### 2.5. TBARS (mg Malonaldehyde/kg)

The modified method from [13] was used to measure TBARS during storage. A 5 g sample of minced beef was combined with 25 mL of a stock solution containing 0.02 M thiobarbituric acid (Sigma Chemical Co., St. Louis, MO, USA) and a 75% trichloroacetic acid mixture with 1% EDTA (Mall-inckrodt Baker Inc., Paris, KY, USA). This mixture was allowed to rest for 20 min at 4 °C, and was then centrifuged at 8000 rpm for 10 min (Rotanta 460R, Hettich, Tuttlingen, Germany). The filtrate was heated in a water bath at 90 °C for 40 min to develop a pink color, and was then cooled. The absorbance of the supernatant was measured spectrophotometrically at 532 nm (μQuant, Microplate Spectrophotometer, Biotek Instruments, Winooski, VT, USA) against a blank without the meat. The TBARS values were reported as mg of MDA per kg of sample, averaged from three beef samples per pretreatment at each sampling time.

### 2.6. pH Value

According to the method by [14], 5 g minced beef meat was homogenized (1 min, 24,000 rpm) in 45 mL of distilled water using the homogenizer (HG-202, K-12S, Hsiang Tai Co., Ltd., New Taipei City, Taiwan). The pH of the beef homogenates was measured at 20 °C using a pH meter (Model PY-P30 Sartorius, Goettingen, Germany). Three samples for each treatment were analyzed at each sampling time, and the results were reported as mean ± SE.

### 2.7. Total Plate Count

According to the method by [15], beef was minced under sterile conditions, and 5 g of the minced meat was aseptically transferred into individual stomacher bags (Seward Medical, Worthing, West Sussex, UK), each containing 45 mL of sterile normal saline (0.9%). The mixture was homogenized in a stomacher (Lab Blender Stomacher 80, Seward Medical, UK) for 2 min. For each sample, the appropriate serial decimal dilutions were prepared in sterile normal saline (0.9%). A volume of 0.1 mL from these serial dilutions of beef homogenates was spread onto the surface of dry media. The total plate count (TPC) was determined using Plate Count Agar (PCA, Merck code 1.05463, Darmstadt, Germany) after incubation for 48 h at 37 °C. Microbial counts were expressed as logarithms of the number of colony-forming units per gram (log10 CFU/g). The TPC values represented the mean of three beef samples per pretreatment at each sampling time.

### 2.8. Salmonella Plate Count

The measurement method is identical to that used for total bacterial count. Specifically, 1 mL of diluted sample was evenly spread onto CHROM agar Salmonella plus (TCH050M, Taiwan Prepared Media Co., Ltd., Taipei City, Taiwan) until absorbed. The plates were then inverted and cultured at 37 ± 0.5 °C for 24 ± 2 h, followed by the counting of the purple-red colonies.

### 2.9. Escherichia coli Count

The measurement method is identical to that used for total bacterial count. Specifically, 1 mL of the diluted sample was evenly spread onto CHROM agar Enterobacteria/ECC (EB042, Taiwan Prepared Media Co., Ltd., Taiwan) until absorbed. The plates were then inverted and cultured at 37 ± 0.5 °C for 24 ± 2 h, followed by colony counting.

### 2.10. Color Values of L*, a*, b*

The modified method from [14] was used to determine the surface color of the beef meat using a colorimeter (Micro Color, Drlange, Germany) from day 0 to day 7. Color measurements were averaged from five readings per sample, with three samples analyzed for each pretreatment at each sampling time. Five CIE Lab* readings were obtained per package. The appearance of the beef sample after the packaging has been opened for the 30 min blooming period was recorded. The chroma meter was standardized against a white tile (CIE L* + 97.56, a* + 0.12, b* + 1.63). The L* value indicates lightness, reflecting total light on a scale from 0 (black) to 100 (white). The a* value represents the red (positive) and green (negative) color balance, while the b* value measures the yellow (positive) and blue (negative) color intensity of the sample.

### 2.11. Metmyoglobin Percentage

The modified method of [16] was used to perform the metmyoglobin (met-Mb) analysis to establish the percentage of total myoglobin in the beef meats. Briefly, 3 g of minced meat was homogenized (HG-202, K-12S, Hsiang Tai Co., Ltd., New Taipei City, Taiwan) in 30 mL of 0.04 M phosphate buffer, at a pH of 6.8. Homogenates were held on ice for 30 min to allow complete pigment extraction before centrifugation (15,000× *g*) for 10 min at 4 °C (Rotanta 460R, Hettich, Germany). The met-Mb (% of total) was calculated based on the absorbance of clarified extract at 525, 572, and 700 nm using the Spectrophotometer (μQuant, Microplate BioTek Instruments, USA). The met-Mb (% of total) was calculated using the following formulas:

met-MB (%) = {1.395 − [(A572 − A700)/(A525 − A700)]} × 100.

The met-Mb values were the mean of three steaks per pretreatment at each sampling time.

### 2.12. Statistical Analysis

The statistical analysis in this study was conducted according to the General Linear Models Procedure of Statistical Analysis System (Version 9.3 for Windows). The differences in average values were compared using one-way ANOVA with Tukey’s honestly significant difference (HSD) test calculator for comparing multiple treatments. A *p* value of less than 0.05 was considered to be significant.

## 3. Results

### 3.1. TBARS (mg Malonaldehyde/kg)

**Figure 1:**
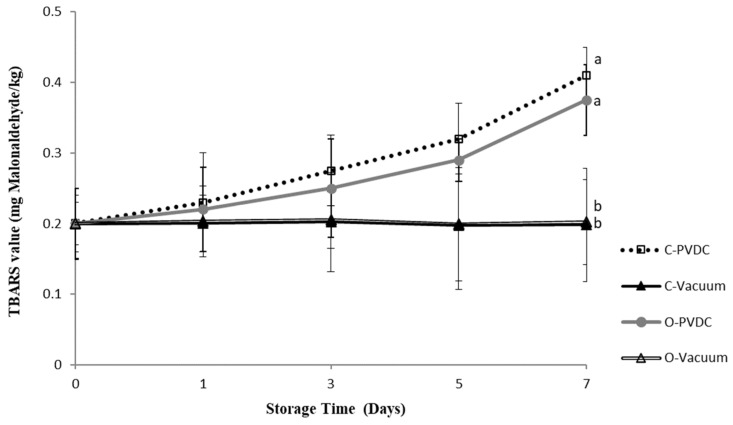
The change in TBARS (mg malonaldehyde/kg) value of the PVDC or Vacuum-packed domestic beef meat round during storage at 4 °C under different treatments. C-PVDC = Control + PVDC with tray packaging, C-Vacuum = Control+ Vacuum packaging, O-PVDC = Ozone water 30 s + PVDC with tray packaging, O-Vacuum = Ozone water 30 s + Vacuum packaging. The data were represented with mean values ± standard error; the different letters (a, b) on the right of the panel mean a significant difference between C-PVDC, C-Vacuum, O-PVDC, and O-Vacuum on the 7th day. Three separate samples of each treatment were measured (n = 3).

Figure 1 presents the results of the TBARS (mg malonaldehyde/kg) value. TBARS values increased with storage duration, reaching their highest point in the C-PVDC group at day 7 (0.41 ± 0.04 mg/kg), followed by the O-PVDC treatment group (0.375 ± 0.05 mg/kg), and the lowest values were observed in both the C-Vacuum treatment group (0.198 ± 0.08 mg/kg) and the O-Vacuum treatment group (0.202 ± 0.06 mg/kg), and these values were significantly different compared to the two PVDC packaging treatment groups (*p* < 0.05).

### 3.2. pH Value

**Figure 2:**
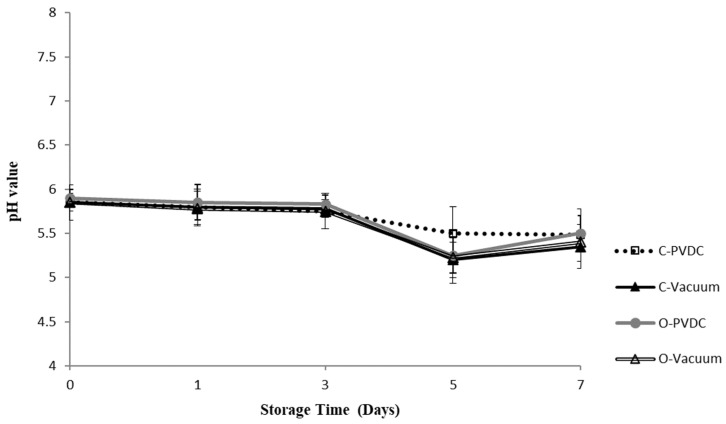
The change in pH value of the PVDC- or Vacuum-packed domestic beef meat rounds during storage at 4 °C under different treatments. C-PVDC = Control + PVDC with tray packaging, C-Vacuum = Control+ Vacuum packaging, O-PVDC = Ozone water 30 s + PVDC with tray packaging, O-Vacuum = Ozone water 30 s + Vacuum packaging. The data were represented with mean values ± standard error. Three separate samples of each treatment were measured (n = 3).

Figure 2 shows the results for the pH value. Throughout the storage period, all treatment groups maintained pH levels between 5.3 and 5.8. There was a downward trend in pH values observed across all treatment groups with increasing storage duration; however, no significant differences were noted among the different treatment groups (*p* > 0.05), indicating that ozone water had no significant impact on the pH values of the beef.

### 3.3. Total Plate Count

**Figure 3:**
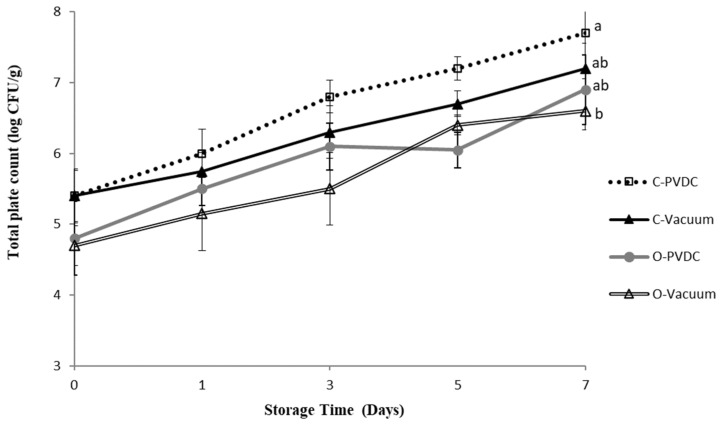
The change in total plate count of PVDC- or Vacuum-packed domestic beef meat rounds during storage at 4 °C under different treatments. C-PVDC = Control + PVDC with tray packaging, C-Vacuum = Control+ Vacuum packaging, O-PVDC = Ozone water 30 s + PVDC with tray packaging, O-Vacuum = Ozone water 30 s + Vacuum packaging. The data were represented with mean values ± standard error; the different letters (a, b) on the right of the panel mean a significant difference in C-PVDC, C-Vacuum, O-PVDC, and O-Vacuum on the 7th day. Three separate samples of each treatment were measured (n = 3).

Figure 3 shows the results for the total plate count. The total plate count increased with storage time in all groups; however, the ozone and vacuum-packaged group (O-Vacuum) exhibited the lowest values (Day 0: 4.7 ± 0.2 Log/g to Day 7: 6.6 ± 0.2 Log/g). By the 7th day, the bacterial count reached 7.7 ± 0.6 Log/g in the Control-PVDC (C-PVDC) group, whereas in the O-Vacuum group, it was significantly lower at 6.6 ± 0.2 Log/g (*p* < 0.05). Both the C-PVDC and C-Vacuum groups showed significantly higher bacterial counts on the 7th day compared to day 0 of storage, whereas the bacterial count in the O-Vacuum group showed an increasing trend with storage time but without significant differences (*p* > 0.05). Fresh meat from slaughterhouses in Taiwan must comply with CAS high-quality meat products certification standards and the veterinary inspection at slaughter by the Animal and Plant Health Inspection Agency, Ministry of Agriculture. The official requirement for microbial total plate count in fresh beef is 7.4 log/g. In this experiment, only the C-PVDC group exceeded the standard on days 5 and 7, while all other samples met the official requirements. The results demonstrate that the use of ozone treatment can improve the problem of microbial counts exceeding the standard in beef packaged with C-PVDC with the storage time.

### 3.4. Salmonella Plate Count

**Figure 4:**
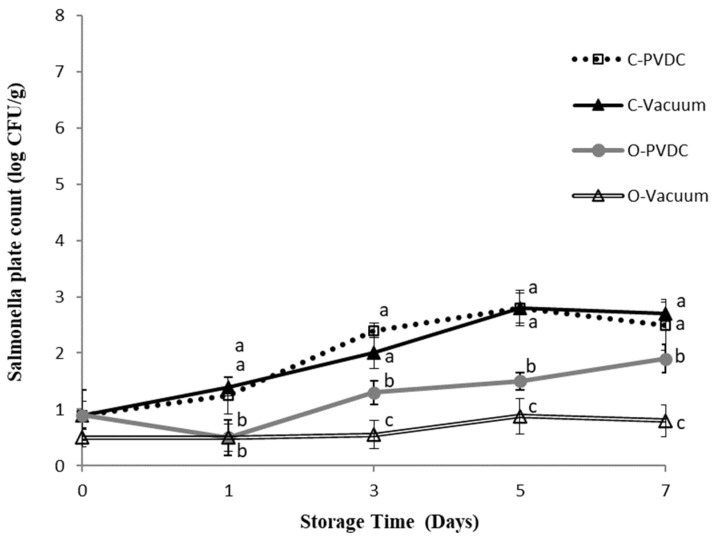
The change in Salmonella plate count of PVDC- or Vacuum-packed domestic beef meat rounds during storage at 4 °C under different treatments. C-PVDC = Control + PVDC with tray packaging, C-Vacuum = Control+ Vacuum packaging, O-PVDC = Ozone water 30 s + PVDC with tray packaging, O-Vacuum = Ozone water 30 s + Vacuum packaging. The aata were represented with mean values ± standard error; the different letters (a–c) on the right of the panel mean a significant difference in C-PVDC, C-Vacuum, O-PVDC, and O-Vacuum at each storage time. Three separate samples of each treatment were measured (n = 3).

Figure 4 shows the results for the Salmonella plate count. In this study, E. coli and Salmonella were not subjected to any specific inoculation procedure; the microbial counts measured were derived from the beef itself sourced from cattle carcasses. We believe this may be attributable to the environment of local commercial slaughterhouses or the equipment used. The Salmonella count in the Control-PVDC (C-PVDC) group was significantly higher than in the two ozone-treated groups (O-PVDC and O-Vacuum) on days 3 and 5 (*p* < 0.05), reaching 2.4 ± 0.1 Log/g to 2.8 ± 0.2 Log/g. For the O-PVDC group, the Salmonella counts were 1.3 ± 0.2 Log/g and 1.5 ± 0.1 Log/g on these respective days. By the 7th day, the growth of Salmonella slowed down, with counts of 2.5 ± 0.4 Log/g and 1.9 ± 0.2 Log/g in the C-PVDC and O-PVDC groups, respectively. The Salmonella count measurements in the O-Vacuum group were significantly (*p* < 0.05) lower than the other three treatment groups on days 1 (0.5 ± 0.1 Log/g), 3 (0.5 ± 0.2 Log/g), 5 (0.8 ± 0.2 Log/g), and 7 (0.8 ± 0.2 Log/g), indicating consistently lower Salmonella counts throughout the storage period compared to the other treatments.

### 3.5. Escherichia coli Count

**Figure 5:**
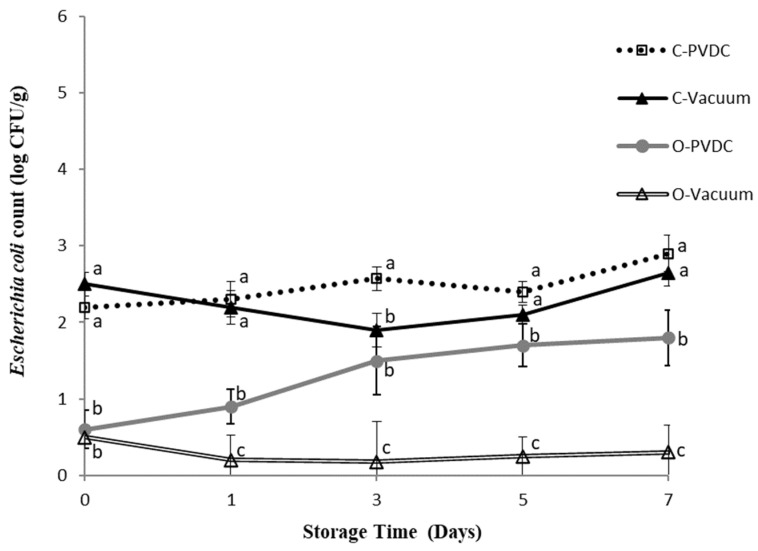
The change in *Escherichia coli* count of PVDC- or Vacuum-packed domestic beef meat rounds during storage at 4 °C under different treatments. C-PVDC= Control + PVDC with tray packaging, C-Vacuum = Control + Vacuum packaging, O-PVDC= Ozone water 30 s + PVDC with tray packaging, O-Vacuum = Ozone water 30 s + Vacuum packaging. The data were represented with mean values ± standard error; the different letters (a–c) on the right of the panel mean a significant difference in C-PVDC, C-Vacuum, O-PVDC, and O-Vacuum at each storage time. Three separate samples of each treatment were measured (n = 3).

Figure 5 shows the results for the *Escherichia coli* plate count. The bacterial count of *Escherichia coli* in each treatment group showed an increasing trend over storage time, but without significant differences (*p* > 0.05). The O-PVDC treatment group exhibited significantly lower counts than the C-PVDC treatment group on days 0 and 3 (*p* < 0.05), and consistently lower counts compared to C-PVDC throughout the storage period. Similarly, the O-Vacuum treatment group consistently showed the lowest counts of *Escherichia coli* throughout the storage period (*p* < 0.05), although again without significant differences as storage time increased. These results demonstrate the effective bactericidal effects of ozone application (1.0~2.0 ppm) in PVDC-tray packaging or vacuum packaging (O-PVDC and O-Vacuum) of domestic beef meat rounds, with bacterial counts one Log lower compared to the two control groups (C-PVDC and C-Vacuum). This finding is consistent with the results of [17], who mixed ground beef with a composite bacterial solution of *Escherichia coli* and Salmonella, treated it with 1% ozone water for 7 or 15 min, and compared the *Escherichia coli*, Salmonella, and anaerobic bacterial counts with other bactericidal water treatments over 0 to 7 days. The results showed that the group treated with ozone water for 15 min significantly reduced all bacteria except *Escherichia coli*, while the group treated with ozone water for 7 min significantly reduced the counts of Salmonella and anaerobic bacteria (*p* < 0.05).

### 3.6. Color Values of L*, a*, b*

**Figure 6:**
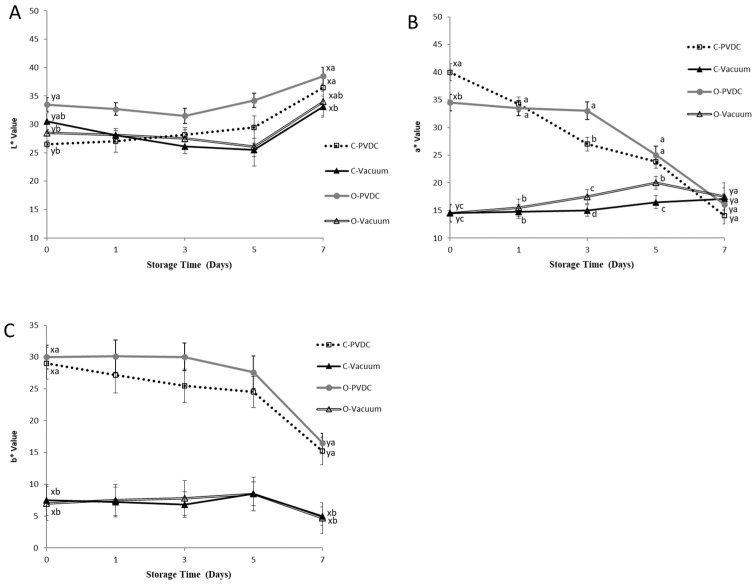
The change in color values of L* (**A**), a* (**B**), and b* (**C**) of PVDC- or vacuum-packed domestic beef meat rounds during storage at 4 °C under different treatments. C-PVDC = Control + PVDC with tray packaging, C-Vacuum = Control+ Vacuum packaging, O-PVDC= Ozone water 30 s + PVDC with tray packaging, O-Vacuum = Ozone water 30 s + Vacuum packaging. The data were represented with mean values ± standard error; the different letters (a–d) on the right of the panel mean a significant difference in C-PVDC, C-Vacuum, O-PVDC, and O-Vacuum at each storage time; the different letters (x, y) on the right of the panel mean a significant difference between day 0 and day 7. Three separate samples of each treatment were measured (n = 3).

Figure 6 shows the results for the color values of L* (A), a* (B), and b* (C). The L* values of all treatment groups (Figure 6A) showed an increasing trend with storage time. When comparing the values between day 0 and day 7 within each treatment group, a significant increase was observed (*p* < 0.05). On day 0, the L* value of the O-PVDC treatment group was the highest among the four treatments. This result is related to the results of the a* values (Figure 6B) on day 0, where the a* value of the O-PVDC treatment group was lower than that of the C-PVDC treatment group (*p* < 0.05). The results for the a* value also found the significant differences between vacuum packaging (C-Vacuum and O-Vacuum) and PVDC packaging (C-PVDC and O-PVDC) at days 0, 1, 3, and 5, this result also indicates that compared to PVDC packaging, vacuum packaging affects the redness of meat not only by reducing the a* value, but also maintains the a* value stability better, due to its sealing properties. But there was no significant difference observed in the b* values (Figure 6C) between the two PVDC treatments on day 0 (*p* > 0.05). As storage time increased, both a* and b* values of the PVDC treatment groups decreased, and there were significant differences between the values on day 7 compared to day 0 (*p* < 0.05). In this experiment, the loss of a* value was not only observed in the ozonized samples; the PVDC treatment group also showed a decrease in redness as storage time increased. However, the vacuum-packaged treatment group had a lower a* value on day 0. The results indicated that in the vacuum-packaged groups, on the 3rd and 5th days, the a* value of the O-Vacuum treatment group was significantly higher than that of the C-Vacuum group. Therefore, the ozone-treated beef in vacuum packaging was able to maintain a higher a* value compared to the untreated beef from days 3 to 5; however, no significant differences were found on day 7.

### 3.7. Metmyoglobin Percentage and Appearance Color Change

**Figure 7:**
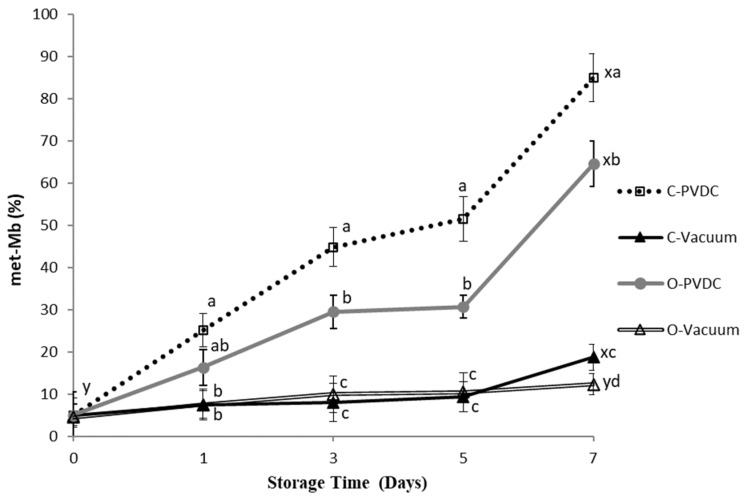
The change in metmyoglobin values (met-Mb percentage) of PVDC- or vacuum-packed domestic beef meat rounds during storage at 4 °C under different treatments. C-PVDC = Control + PVDC with tray packaging, C-Vacuum = Control+ Vacuum packaging, O-PVDC = Ozone water 30 s + PVDC with tray packaging, O-Vacuum = Ozone water 30 s + Vacuum packaging. The data were represented with mean values ± standard error; the different letters (a–d) on the right of the panel mean a significant difference in C-PVDC, C-Vacuum, O-PVDC, and O-Vacuum at each storage time; the different letters (x, y) on the right of the panel mean a significant difference between day 0 and day 7. Three separate samples of each treatment were measured (n = 3).

The color of fresh meat is determined by the relative concentrations of three myoglobin derivatives [18]. Deoxymyoglobin (DOMb) and oxymyoglobin (MbO2), both in their reduced forms, can oxidize to form metmyoglobin (met-Mb), which is characterized by a dull brown hue that is indicative of quality deterioration [19]. Figure 7 shows the results for the Metmyoglobin percentage. The metmyoglobin content increased in all treatment groups with storage time, and the values on the 7th day were significantly higher than those on the 0th day (*p* < 0.05). Specifically, on the 3rd, 5th, and 7th days, the metmyoglobin content in the O-PVDC treatment group was significantly lower than that in the C-PVDC control group (*p* < 0.05). This could be attributed to the rapid degradation of ozone molecules into odorless and pollution-free oxygen, which, upon binding with hemoglobin, inhibits the formation of denatured metmyoglobin [20]. Under vacuum packaging conditions, there was no significant difference between the 0th and 5th days, regardless of ozone treatment (*p* > 0.05); however, on the 7th day, the metmyoglobin content in the O-Vacuum treatment group was significantly lower than that in the C-Vacuum control group (*p* < 0.05), which is consistent with the results observed in both PVDC-trap and vacuum packaging.

**Figure 8:**
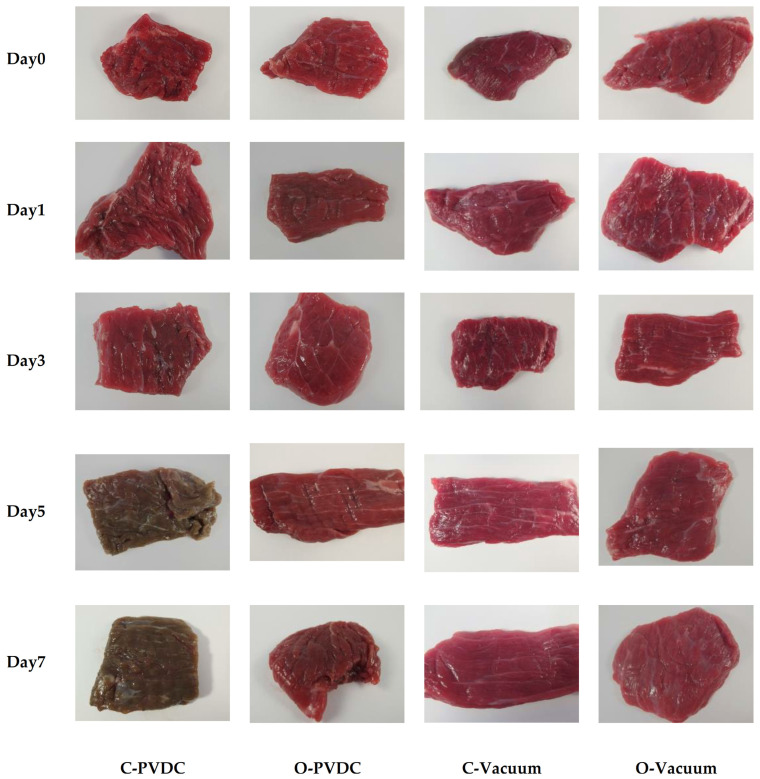
The appearance color change in PVDC- or vacuum-packed (photo taken after a 30 min blooming period following the opening of the packaging) domestic beef meat rounds during storage at 4 °C under different treatments. C-PVDC = Control + PVDC with tray packaging, C-Vacuum = Control+ Vacuum packaging, O-PVDC = Ozone water 30 s + PVDC with tray packaging, O-Vacuum = Ozone water 30 s + Vacuum packaging.

The above results can be corroborated by the changes in the color appearance of the domestically produced beef under different treatments during storage, as shown in Figure 8. From left to right, the images represent the C-PVDC, O-PVDC, C-Vacuum, and O-Vacuum treatment groups, while from top to bottom, they represent storage durations of 0, 3, 5, and 7 days. On the 0th day, the beef color in the O-PVDC treatment group was lighter, consistent with the results of L* and a* values. By the 5th day of storage, the color of the C-PVDC treatment group turned brown, correlating with the changes in denatured metmyoglobin content. Even on the 7th day of storage, the color of the beef in the O-PVDC treatment group remained close to the fresh beef color, which is associated with the significantly lower metmyoglobin content in the O-PVDC treatment group compared to the C-PVDC control group (*p* < 0.05).

## 4. Discussion

As for why this study uses ozone in its aqueous form instead of gas, research indicates that both forms can be used [5], each with its advantages. Ozone water is more widely used in the food processing field than ozone gas because (1) it convenient to use and easy to operate, especially in food processing and washing; (2) it can directly contact food items for surface disinfection, reducing microbial load; (3) it effective for cleaning food contact surfaces and equipment. This study aims to assess the feasibility of applying it in beef processing plants to improve meat hygiene, so using ozone water is easier for staff to operate compared to gas, especially as the gas form requires special safety measures to prevent excessive exposure to ozone gas during its use by staff. Both gaseous and aqueous forms of ozone are effective choices for food safety and hygiene. The specific application should be determined based on actual needs and the operational environment to achieve optimal disinfection results.

The TBARS value variations during storage for 0 to 7 days in the two vacuum packaging groups revealed no significant differences between the treatment groups (*p* > 0.05). Additionally, there were no significant differences observed when comparing each treatment group between day 0 and day 7 (*p* > 0.05). This outcome may be attributed to the removal of a significant portion of air in vacuum packaging, which prevents beef oxidation due to oxygen exposure. Packaging beef meat protects it against deterioration. Vacuum packages lacking O_2_ can minimize the oxidative deteriorative reactions [10], indicating the effective stabilization of meat or meat products during storage with vacuum packaging. Ockerman and Kuo [21] indicated that noticeable off-flavors from lipid oxidation occur when TBARS values exceed 1.0. Another study also said that taking into account that values of up to 0.6 mg of malondialdehyde kg^−1^ of fresh meat are considered acceptable [22], it can be concluded that the meat treated with ozone water for 30 s showed no significant oxidation. The TBARS results indicated no significant differences (*p* > 0.05) between the control (C-Vacuum) and the ozone-treated samples (O-Vacuum). In contrast, a 24 h ozonation treatment resulted in a malondialdehyde level of 0.96 mg kg−1 of fresh meat at 0 °C, which is deemed unacceptable for consumption [23]. In the present study, the TBARS values for both packaging methods during storage did not exceed 0.5 mg/kg, and the results demonstrate that beef treated with ozone in conjunction with vacuum packaging effectively inhibits the increase in TBARS values during one week of refrigerated storage, thereby preventing oxidative deterioration.

The pH value results align with the findings of [24], who investigated the effects of different volume ratios of carbon monoxide and ozone on beef subjected to vacuum and modified-atmosphere packaging, stored at 0 °C for 46 days. Their study reported an initial pH range of 5.59–5.73, with no significant differences observed among the groups at baseline. Notably, the pH of all steaks decreased significantly during the early stages of storage. Similar observations have been documented in other studies [25,26]. Two primary factors contributed to this phenomenon. First, the contraction of muscle fibers was dependent solely on energy produced through glycolysis, due to the absence of oxygen, resulting in the accumulation of lactate and pyruvate. Second, the degradation of ATP generated phosphoric acid. These acidic conditions facilitated the softening and expansion of connective tissue, both within and outside the muscle membrane, as well as collagen fibers, thereby consuming the acidic medium [27]. Stivarius et al. [17] compared the pH values of ground beef mixed with bacterial solution and soaked in 1% ozone water for 7 or 15 min with other sterilization treatments during storage for 0 to 7 days, finding no significant differences (*p* > 0.05) in pH values among treatment groups.

Total plate count estimation is commonly used as an index for the acceptability criteria, guidelines, and specifications for fresh beef [24]. Spoilage is assessed by measuring the total plate count on the beef. Spoilt beef typically has a count > 7.00 Log CFU/g [28], in this experiment, only the C-PVDC group exceeded the standard on days 5 and 7, the other treatment groups, due to the ozone treatment or vacuum packaging, had total plate counts within acceptable ranges up to day 7. This study analyzed the effects of gaseous ozone treatment at refrigeration temperatures on microbial counts, specifically total aerobic mesophilic heterotrophic microorganisms and inoculated *Escherichia coli*, in the culture media and beef samples [23]. Additionally, the influence of ozone on beef quality properties, such as surface color and lipid oxidation (measured by TBARS), was assessed. The application of gaseous ozone (154 × 10^−6^ kg m^−3^) to culture media inoculated with *E. coli* resulted in total inactivation of the microorganism after 3 or 24 h of treatment at 0° and 4 °C. In beef samples subjected to the same ozone concentration, the highest level of microbial inhibition occurred at 0 °C after 24 h, leading to decreases of 0.7 log_10_ cycles in *E. coli* counts and 2.0 log_10_ cycles in total aerobic mesophilic heterotrophic microorganisms. However, the surface color and lipid oxidation of these beef samples remained unacceptable. Shorter exposure times (3 h) to the ozone concentration at both 0 °C and 4 °C resulted in a reduction of 0.5 log_10_ cycles in total aerobic mesophilic heterotrophic microorganisms and a decrease of 0.6 to 1.0 log_10_ cycles in *E. coli* counts, without affecting the color of or causing rancidity in the beef.

Another study was conducted by [24] that investigated beef samples without pretreatment (CK) and those pretreated with various volume ratios of carbon monoxide and ozone: 100% CO (T1), 2% O3 + 98% CO (T2), 5% O3 + 95% CO (T3), and 10% O3 + 90% CO (T4). These samples were exposed to modified atmosphere packaging for 1.5 h, followed by vacuum packaging and storage at 0 °C for 46 days. The ozone pretreatment significantly reduced the colony counts in T2, T3, and T4, with the largest reductions observed in T3 and T4, decreasing from 4.93 log_10_ CFU/g to 4.08 and 3.90 log_10_ CFU/g, respectively, on day 0. Ozone demonstrated effective sterilization capabilities that correlated with its concentration. Furthermore, ozone treatment was reported to reduce counts by 1.3 log_10_ CFU/g on beef carcasses compared to untreated samples [29]. Throughout most of the storage period, T3 and T4 exhibited greater sterilization effectiveness than T2, likely due to their higher ozone concentrations [24]. Although another study also uses aqueous ozone (5 ppm O_3_ for 5 min) and then vacuum-packaged the samples to 2 kPa for 10 days storage at 37 °C, 25 °C, or 4 °C [30], it focused on the impact on *clostridium perfringens*, and its results also found that vacuum packaging at 37 °C resulted in spore germination, outgrowth, and rapid proliferation to levels exceeding 7 log^10^ cfu/g of beef after storage, and indicated that the ozone treatment of spores on beef surfaces usually achieves a reduction of about 1 log10 cfu/g in initial inoculum levels; a key aspect of prefabrication treatment is maintaining constant refrigeration temperatures, which also improves the efficiency of ozone pretreatment.

In beef tissues, color is a critical factor influencing consumer acceptability [17]. The L* values of all treatment groups (Figure 6A) showed an increasing trend with storage time. When comparing the values between day 0 and day 7 within each treatment group, a significant increase was observed (*p* < 0.05). This could be attributed to the gradual denaturation and breakdown of muscle proteins over time, leading to a reduction in their water-holding capacity. Consequently, surface water infiltration occurred, increasing brightness values [31]. The beef samples were packaged using either PVDC or vacuum packaging after being treated or not treated with ozone, and then color measurements were taken. So, the results showed the color of the beef sample in the different groups differed at day 0, it is hypothesized that the vacuum packaging immediately reduces the exposure of beef to oxygen, which affects the a* and b* values on the surface of the meat at the beginning of the experiment. On day 0, the L* value of the O-PVDC treatment group was the highest among the four treatments. This result is related to the results of the a* values (Figure 6B) on day 0, where the a* value of the O-PVDC treatment group was lower than that of the C-PVDC treatment group (*p* < 0.05). This could be attributed to ozone being a strong oxidizing agent, which alters the chemical state of myoglobin in the muscle upon contact with the meat, causing discoloration and resulting in an increase in L* values and a decrease in a* values. As storage time increased, both the a* and b* values of the PVDC treatment groups decreased, and there were significant differences between the values on day 7 compared to day 0 (*p* < 0.05).

Brewer et al. [11] indicated that when myoglobin rapidly comes into contact with oxygen in the air to form oxymyoglobin, the a* value significantly increases. In the case of the vacuum-packaged groups, on the 3rd and 5th days, the a* value of the O-Vacuum treatment group was significantly higher than that of the C-Vacuum group without ozone treatment (*p* < 0.05). This may be attributed to ozone being a strong oxidizing agent, causing the myoglobin to undergo chemical changes leading to the discoloration of the beef. Additionally, in vacuum packaging where oxygen is lacking, meat tends to exhibit a darker purplish-red color compared to the bright cherry-red color. The combination of these factors results in the ozone-treated groups having higher a* values measured in vacuum packaging bags. The color values (L*, a*, b*) of this storage experiment are similar to those of [17], who conducted a study involving ground beef treated with a 1% concentration of ozone water immersion for 7 or 15 min after inoculation with a mixed bacterial culture. They compared the microbial content and color analysis with other antimicrobial water treatments over 0 to 7 days. The results showed that with increasing storage time, the L* values of all treatment groups exhibited an upward trend, while the a* and b* values of each treatment group gradually decreased. The L* values of the vacuum-packaged and PVDC with tray-packaged samples ranged between approximately 30 and 40. Although the L* values of the two vacuum-packaged treatment groups were lower than those of the two PVDC-packaged treatment groups (Figure 6A), there was no significant difference (*p* > 0.05), indicating that the L* values did not vary significantly due to different packaging methods. This result may be attributed to the primary function of vacuum packaging, which is to reduce the probability and capacity of oxygen oxidation on meat products. Its main effect on meat products is on the coloration ability of pigment proteins in the muscle. Therefore, it has no significant impact on the L* values representing brightness or darkness [11].

The parameter a* decreased in comparison to the control sample, due to the oxidation of myoglobin and oxymyoglobin to metmyoglobin. This decrease is linked to the loss of the oxymyoglobin pigment. These findings are in agreement with [32] and [17]; who reported similar reductions in a* values for rib-eye steaks treated with ozone over extended storage periods. It should be noted that, according to the literature, in the case of refrigerated beef, redness values of a* > 14 are acceptable to consumers [33]. Fournaud and Lauret [34] noted that ozone treatment negatively impacted beef color, while Kaess and Weidemann [35] found that low ozone concentrations had minimal effects on color. As shown in Figure 7, the met-Mb levels in both pretreated and untreated samples increased during storage. This observation aligns with the findings of [36], suggesting that the rise in met-Mb levels may be attributed to the declining activity of the met-Mb-reducing enzyme system [37]. With the extension of the storage period, the samples displayed a significant difference between the vacuum groups (control/ozone) and the PVDC groups of control and ozone. On longer storage, C-vacuum and O-vacuum groups showed the lowest met-Mb, followed by O-PVDC and C-PVDC. Many factors affect the formation of met-Mb, including met-Mb-reducing enzyme activity, oxygen pressure, lipid oxidation, and so forth [38]. The oxidation of lipids could enhance myoglobin oxidation due to the reactivity of primary and secondary products derived from unsaturated fatty acids [39]. From the results of Figure 1 and Figure 7, TBARS values of the groups packaged with vacuum were significantly lower than C-PVDC and O-PVDC groups on longer storage. Therefore, the lower lipid oxidation of C-Vacuum and O-Vacuum might result in lower met-Mb as compared with the PVDC groups.

Generally, longer storage for shelf-life tests (more than 7 days) could be conducted to evaluate the impact of ozone combined with vacuum packaging on the shelf life of beef. However, as we mentioned in the introduction, fresh domestically produced Taiwanese beef has a specific consumer market, and this beef is not frozen. The demand from consumers is for freshness from farm to table on the same day after slaughter, so it is typically consumed within a week. Therefore, we focused on experiments within that week.

## 5. Conclusions

Based on the experimental results presented, it is evident that ozone treatment, especially when combined with vacuum packaging, significantly inhibited microbial growth in domestically produced beef meat rounds during refrigerated storage. This was demonstrated by lower TBARS values and bacterial counts in the ozone-treated groups compared to the control groups. Additionally, relative to the differences in packaging methods, ozone treatment has a smaller effect on the pH, color, and lipid oxidation of beef samples. Ozone-treated beef in vacuum packaging was able to maintain a higher a* value compared to untreated beef. The combination of ozone treatment and vacuum packaging showed promising potential in preserving beef quality and enhancing food safety.

## Data Availability

The original contributions presented in the study are included in the article, further inquiries can be directed to the corresponding author.
